# Flavonoids from *Artemisia annua* L*.* as Antioxidants and Their Potential Synergism with Artemisinin against Malaria and Cancer

**DOI:** 10.3390/molecules15053135

**Published:** 2010-04-29

**Authors:** Jorge F.S. Ferreira, Devanand L. Luthria, Tomikazu Sasaki, Arne Heyerick

**Affiliations:** 1 USDA-ARS, Appalachian Farming Systems Research Center, 1224 Airport Rd., Beaver, WV 25813, USA; 2 USDA-ARS, Food Composition and Methods Development Lab, 10300 Baltimore Ave,. Bldg 161 BARC-East, Beltsville, MD 20705-2350, USA; E-Mail: D.Luthria@ars.usda.gov (D.L.L.); 3 Department of Chemistry, Box 351700, University of Washington, Seattle, WA 98195-1700, USA; E-Mail: sasaki@chem.washington.edu (T.S.); 4 Laboratory of Pharmacognosy and Phytochemistry, Ghent University, Harelbekestraat 72, B-9000 Ghent, Belgium; E-Mail: Arne.Heyerick@UGent.be (A.H.)

**Keywords:** *Artemisia annua*, artemisinin, flavonoids, antimalarial, anticancer, synergism

## Abstract

*Artemisia annua* is currently the only commercial source of the sesquiterpene lactone artemisinin. Since artemisinin was discovered as the active component of *A. annua* in early 1970s, hundreds of papers have focused on the anti-parasitic effects of artemisinin and its semi-synthetic analogs dihydroartemisinin, artemether, arteether, and artesunate. Artemisinin per se has not been used in mainstream clinical practice due to its poor bioavailability when compared to its analogs. In the past decade, the work with artemisinin-based compounds has expanded to their anti-cancer properties. Although artemisinin is a major bioactive component present in the traditional Chinese herbal preparations (tea), leaf flavonoids, also present in the tea, have shown a variety of biological activities and may synergize the effects of artemisinin against malaria and cancer. However, only a few studies have focused on the potential synergistic effects between flavonoids and artemisinin. The resurgent idea that multi-component drug therapy might be better than monotherapy is illustrated by the recent resolution of the World Health Organization to support artemisinin-based combination therapies (ACT), instead of the previously used monotherapy with artemisinins. In this critical review we will discuss the possibility that artemisinin and its semi-synthetic analogs might become more effective to treat parasitic diseases (such as malaria) and cancer if simultaneously delivered with flavonoids. The flavonoids present in *A. annua* leaves have been linked to suppression of CYP450 enzymes responsible for altering the absorption and metabolism of artemisinin in the body, but also have been linked to a beneficial immunomodulatory activity in subjects afflicted with parasitic and chronic diseases.

## 1. Introduction

A brief search through PubMed, on March 2010, using the keywords “artemisinin” and “malaria” returned 1,266 hits, while using “*Artemisia annua*” and “flavonoids” returned 12 hits, but “artemisia flavonoids” and “*Artemisia annua* flavonoids” combined with “malaria” returned only four and two hits, respectively. In the same way, “artemisinin” and “cancer” returned 117 hits, “Artemisia flavonoids” and “cancer”, 12 hits, and “*Artemisia annua* flavonoids” and “cancer”, only one hit. This search meant to establish that in the past 15 years there has been plenty of research on the activity of artemisinin against malaria, followed by less on cancer. However, much less work has focused on the role of flavonoids from *A. annua* against malaria and cancer. Although “flavonoids” and “cancer” returned 8,420 hits, the search for “flavonoids” and “malaria” returned only 68 hits, indicating that the beneficial effects of flavonoids in cancer prevention are well accepted, but not so much for their involvement in the treatment of malaria. While the potential synergistic effect of flavonoids with artemisinin or other anticancer and antimalarial drugs is far from fully explored, it seems worthwhile to investigate biological interactions of flavonoids and artemisinin derivatives in both malaria and cancer.

In the light of recent reports of *Plasmodium*-resistant strains in Cambodia/Vietnam borders and the shortage of artemisinin as a raw material to produce artemisinin-based combination therapies (ACT), there is a pressing need to increase effectiveness and affordability of artemisinin derivatives and ACT. The combined use of flavonoids with artemisinins might increase effectiveness of artemisinins, but the combination of artemisinins with pyrimethamine, sulfadoxine, and lumefantrine, recommended by the World Health Organization in current ACT, would still be needed to circumvent malaria recrudescence issues. It is known that flavonoids chelate metals such as iron and copper as part of their antioxidant effects and that iron chelating therapies have been recommended for malaria patients [1]. Thus, the use of flavonoids in combination with artemisinin might provide a more effective treatment for malaria. In that regard, flavonoids could serve as artemisinin synergists by reacting with iron and converting Fe^+3^ to Fe^+2^ [2], the latter being important in the bioactivity of artemisinin [3], leading to the release of short-lived toxic free radicals that are part of the antimalarial and anticancer mode of action of artemisinin. 

Individuals afflicted with malaria and cancer have increased blood free radicals [4,5], possibly aggravating the disease scenario or leading to the generation of the disease in the case of cancer. Thus, it might make sense to combine antioxidant flavonoids, tannins, phenolic acids, and coumarins with artemisinins to treat malaria and cancer, as well as to prevent the latter. The *A. annua* traditional tea is a rich source of both antioxidant phenolics (mostly flavonoids) and artemisinin [6,7]. Levels of artemisinin are lower in such teas than in current treatments with ACT and use of the tea [7,8], or artemisinin alone [9], leads to recrudescence levels that vary greatly, from as low as 10%, in the case of a seven-day course with artesunate [10] to 46%–80% in non-immune patients in Thailand and China [11]. This varying recrudescence is not only related to the short half-life of artemisinin, but also to the duration of treatment and to the loss in sensitivity to dihydroartemisinin (the active blood metabolite) by different strains of *Plasmodium*, which is remediated by the combination of artemisinin with other antimalarial drugs of longer half-lives and different modes of action [11]. However, the flavonoids could be the reason why the tea “reportedly” treated malaria for hundreds of years and, although recrudescence might have occurred then as well, there was compelling evidence from traditional Chinese herbal medicine to justify the addition of *A. annua* to the selection of Chinese plants screened for malaria in 1969, which eventually led to the discovery of artemisinin [12]. Although we do not recommend substitution of the tea for the WHO-recommended ACT, the tea might still be valuable in remote areas of Africa to delay malaria-induced coma and allow one to get to a hospital and receive proper treatment.

This review will focus on the flavonoids found in *A. annua* (Table 1) wherever they relate to anticancer or antimalarial effects, on their own or in synergism with other natural compounds, with synthetic anticancer and antimalarial drugs, and with artemisinins. However, one should keep an open mind and complete the idea where he/she judges we failed. No review is final and, based on what in currently known, or strictly based on the chemical structure of flavonoid, it is quite hard to predict the full spectrum of their biological activity. If there would be no biological activity or benefit for flavonoids, hydroxylated or methoxylated, glycosylated or not, why would plants go through such an energetically-expensive endeavor to produce so many different kinds of flavonoids?

## 2. Classification of Plant Phenolics

Phenolic phytochemicals (phenolics) occupy a unique position in the area of natural products due to their ubiquitous distribution throughout the plant kingdom and in products (fruits, vegetables, beverages, herbs, cosmetics and nutraceuticals) consumed by the general population on a regular basis [13]. Phenolics are biosynthesized by plants during normal development and in response to stress conditions such as exposure to UV radiation, pest attack, and wounding [14,15]. Phenolic compounds are known to provide protection against a wide range of diseases such as coronary heart disease, stroke, and certain types of cancers [16,17]. Chemically, phenolics are defined as a class of aromatic organic compounds with at least one hydroxyl group attached directly to a benzene ring [18]. Over 8000 phenolics with wide structural diversity and polarities have been isolated from plants [19]. 

**Figure 1:**
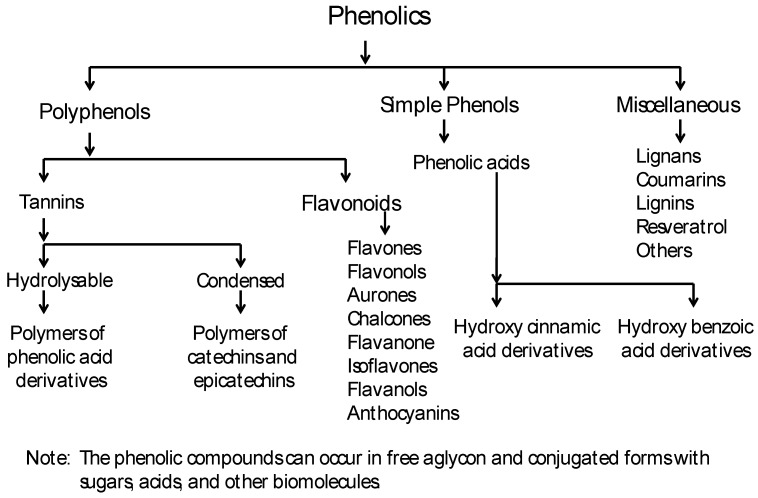
General classification of plant phenolics, modified from [18].

Phenolics can be chemically grouped into three broad categories: polyphenols (tannins and flavonoids), simple phenols (phenolic acids) and a miscellaneous group (Figure 1) [18]. Although we have used this classification, based on chemical structure, for this manuscript, the commonly used byosynthetical classification described in most reviews is also available at http://www.phenol-explorer.eu/compound_classes. Phenolic acids are chemically defined as carboxylic acid derivatives of phenols, whereas no such clear definition for polyphenols is provided in the literature. Rather, polyphenols are described as a group of chemical substances found in plants, characterized by the presence of more than one phenol unit or building block per molecule. Polyphenols serve as antioxidants as they tend to prevent or neutralize the damaging effects of free radicals. They also give flowers, fruits, and vegetables their color. 

Polyphenols can be arranged into two broad classes: tannins and flavonoids. Tannins are astringent, bitter plant polyphenols that either bind or precipitate proteins. Tannins can be further classified chemically into two main groups, hydrolyzable and condensed. Hydrolyzable tannins decompose in water yielding various water-soluble products, such as gallic acid or ellagic acid, protocatechuic acid and sugars. Condensed tannins, also known as proanthocyanidins, are polymers of 2 to 50 (or more) flavonoid units joined by carbon-carbon bonds, which are not cleaved by hydrolysis. Flavonoid is a general name for phytochemicals based on a 15 carbon (C6-C3-C6) skeleton. Over 4,500 different flavonoids have been isolated and identified from plants [20]. Flavonoids can be further divided into multiple groups such as flavones, flavonols, flavanones, dihydroflavonols, chalcones, aurones, isoflavonoids, biflavonoids, *etc.* Flavonoids can occur as free aglycons or as conjugated forms with methoxyl, glycosyl, isoprenyl, prenyl, methylenedioxy, aliphatic acids and other substituents [18,21,22]. 

Phenolic acids on the other hand can be broadly grouped into two subgroups: hydroxylcinnamic and hydroxylbenzoic acids derivatives (Figure 1). In many cases, the aldehyde analogs such as vanillin are also grouped with phenolic acids. The miscellaneous group comprises all other phenolic compounds not classified into the distinct subgroups above. These include lignans, lignins, coumarins, stilbenes derivatives like resveratrol, and other phenolic compounds [18]. Phenolic compounds can also occur as free aglycons or conjugated with one or more substituents such as methoxyl, glycosyl, prenyl, methylenedioxy, aliphatic acids, *etc.*

## 3. Phenolics from *A. annua*

**Figure 2:**
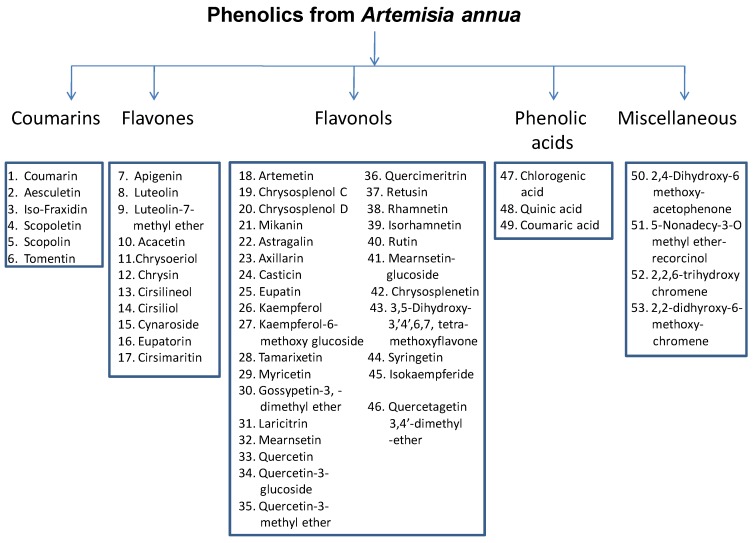
Major phenolics from *Artemisia annua*, with the great majority being flavones or flavonols.

A few recent reports indicate that *A. annua* is one of the four medicinal plants with the highest ORAC (oxygen radical absorbance capacity) level [23,24]. The total antioxidant capacity (ORAC) of *A. annua* leaves and inflorescences extracts was reported as 1,125 and 1,234 µmoles of Trolox equiv/g, respectively, which is half to two thirds of the ORAC of oregano (the highest reported ORAC for an herb) extracts. The high antioxidant activity of *A. annua* extract is most likely due to its high phenolic content. Over 50 different phenolic compounds belonging to five major groups (flavones, flavonols, coumarins, phenolic acids, and a miscellaneous group have been reported from *A. annua *(Figure 2). The prominent coumarins identified from *A. annua* are coumarin, aesculetin (6,7-dihydroxycoumarin), iso-fraxidin (7-hydroxy-6,8-dimethoxycoumarin), scopoletin (7-hydroxy-6-methoxycoumarin), scopolin (7-β-D-glucopyranoside-6-methoxycoumarin), and tomentin (5-hydroxy-6,7-dimethoxy-coumarin). The main components of *A. annua* were recently identified by HPLC-MS as quercetin-glucoside, flaviolin, rhamnetin, chrysoplenol D, and pilloin, although the HPLC-UV data suggested that when detection was done at 335 nm more than 40 components, including chlorogenic acid, were present [25]. The structures of the 11 prominent flavones and 29 flavonols reported from *A. annua* are reported in Table 1 [25,26]. A highly specific feature of *A. annua* is the presence of significant quantities of structurally diverse polymethoxylated flavonoids [27,28]. In addition, other phenolic compounds such as 2,4-dihydroxy-6-methoxy-acetophenone, 5-nonadecyl-3-O-methyletherresorcinol, 2,2,6-trihydroxychromene, and 2,2-dihydroxy-6-methoxychromene have also been isolated from *A. annua* [26].

**Table 1:** Major phenolics reported from *Artemisia annua* and the general structure of flavonoids. The number refers to the number given to each compound in Figure 2. Compounds with a 3-OH group attached to the 2,3-double bond, and adjacent to the 4-carbonyl group in the C ring are predicted to have major antioxidant activity [29]. Substituents (R) are numbered according to the ring position (A, C, and B). 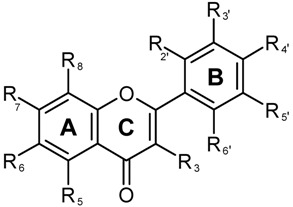

Structure	Phenolic type	Ring and substituent position									
No.	Flavones	C(R_3_)	A(R_5_)	A(R_6_)	A(R_7_)	A(R_8_)	B(R_2’_)	B(R_3’_)	B(R_4’_)	B(R_5’_)	B(R_6’_)
**7**	Apigenin	H	OH	H	OH	H	H	H	OH	H	H
**8**	Luteolin (5,7,3’,4’-Tetrahydroxy flavone)	H	OH	H	OH	H	H	OH	OH	H	H
**9**	Luteolin-7-methylether	H	OH	H	OCH_3_	H	H	OH	OH	H	H
**10**	Acacetin (apigenin-4’-methyl ether) or 5,7-dihydroxy-4-methoxy flavone	H	OH	H	OH	H	H	H	OCH_3_	H	H
**11**	Chrysoeriol (Lutoelin-3’-methyl ether) or5,7,4’-Trihydroxy-3’-methoxy flavone	H	OH	H	OH	H	H	OCH_3_	OH	H	H
**12**	Chrysin (5,7-Dihydroxy flavone)	H	OH	H	OH	H	H	H	H	H	H
**13**	Cirsilineol (6-Hydroxyluteolin-6,7,3’-trimethyl ether or 5,4’-dihydroxy-6,7,3’-trimethoxyflavone, Fastigenin, Anisomelin, Eupatrin)	H	OH	OCH_3_	OCH_3_	H	OH	OCH_3_	OH	H	H
**15**	Cynaroside (Luteolin-7-glucoside or 5,7,3’,4’-Tetrahydroxyflavone-7-glucoside or Glucoluteolin or Luteoloside or Cinaroside)	H	OH	H	OGlu	H	H	OH	OH	H	H
**16**	Eupatorin (6-Hydroxyluteolin-6,7,4’-trimethyl ether or 5,3’-Dihydroxy-6,7,4’-trimethoxyflavone)	H	OH	OCH_3_	OCH_3_	H	H	OH	OCH_3_	H	H
**17**	Cirsimaritin (Scutellarin-6,7-dimethyl ether or 6-Hydroxyapigenin-6,7-dimethyl ether or 5,4’-Dihydroxy-6,7-Dimethoxyflavone or Scorphulein or Cirsumaritin or Cirsitakaogenin)	H	OH	OCH_3_	OCH_3_	H	H	H	OH	H	H
**18**	Artemetin	OCH3	OH	OCH_3_	OCH_3_	H	H	OCH_3_	OCH_3_	H	H
**19**	Chrysosplenol-C	OCH3	OH	OH	OCH_3_	H	H	OCH_3_	OH	H	H
**20**	Chrysosplenol-D	OCH_3_	OH	OCH_3_	OCH_3_	H	H	OH	OH	H	H
**21**	Mikanin	OH	OH	OCH_3_	OCH_3_	H	H	H	OCH_3_	H	H
**22**	Astragalin (Kaempferol-3-α-D-glucoside)	O-glu	OH	H	OH	H	H	H	OH	H	H
**23**	Axillarin (5,7,3’,4’-Tetrahydroxy-3,6-dimethoxyflavone or quercetagetin -3,6- dimethyl ether)	OCH_3_	OH	OCH_3_	OH	H	H	OH	OH	H	H
**24**	Casticin (5,3’-dihydroxy-3,6,7,4’-tetramethyl ether flavone or Quercetagetin -3,6-7,4’-tetramethyl ether)	OCH_3_	OH	OCH_3_	OCH_3_	H	H	OH	OCH_3_	H	H
**25**	Eupatin (3,5,3’-Trihydroxy-6,7,4’-trimethoxyflavone or Quercetagetin -3,6- dimethyl ether)	OH	OH	OCH_3_	OCH_3_	H	H	OH	OCH_3_	H	H
**26**	Kaempferol (3,5,7,4’-Tetrahydroxy flavone)	OH	OH	H	OH	H	H	H	OH	H	H
**27**	Kaempferol-6-methox-3-O-β-D-glucoside	OGlu	OH	OCH_3_	OH	H	H	H	OH	H	H
**28**	Tamarixetin	OH	OH	H	OH	H	H	OH	OCH_3_	H	H
**29**	Myricetin (3,5,7,3’,4’,5’-Hexahydroxy flavone)	OH	OH	H	OH	H	H	OH	OH	OH	H
**30**	Gossypetin- 3,8-dimethylether	OCH_3_	OH	H	OH	OH	H	OH	OCH_3_	H	H
**31**	Laricitrin (3,5,7,3’,4’, -Pentahydroxy 5’-methoxyflavone)	OH	OH	H	OH	H	H	OH	OH	OCH_3_	H
**32**	Mearnsetin (3,5,7,3’,5’, -Pentahydroxy 4’-methoxyflavone or Myricetin-4-methyl ether)	OH	OH	H	OH	H	H	OH	OCH_3_	OH	H
**33**	Quercetin (3,5,7,3’,4’-Pentahydroxy flavone)	OH	OH	H	OH	H	H	OH	OH	H	H
**34**	Quercetin-3’- O-β-D-glucoside	OH	OH	H	OH	H	H	O-Glu	OH	H	H
**35**	Quercetin-3- methylether	OCH_3_	OH	H	OH	H	H	OH	OH	H	H
**36**	Quercimeritrin (Quercetin-7-glucoside)	OH	OH	H	O-Glu	H	H	OH	OH	H	H
**37**	Retusin (5-Hydroxy-3,7,3’4’-tetramethoxy flavone or Quercetin3,7,3’,4’-tetramethylether)	OCH_3_	OH	H	OCH_3_	H	H	OCH_3_	OCH_3_	H	H
**38**	Rhamnetin (Quercetin-7-methylether or 3,5,7,3’-Tetrahydroxy-4’-methoxy flavone)	OH	OH	H	OCH_3_	H	H	OH	OH	H	H
**39**	Isorhamnetin (Quercetin-3’-methylether or 3,5,7,4’-Tetrahydroxy-3’-methoxy flavone)	OH	OH	H	OH	H	H	OCH_3_	OH	H	H
**40**	Rutin (Quercetin-3-rutinoside)	O-Diglyc.	OH	H	OH	H	H	OH	OH	H	H
**41**	Mearncetin glucoside	OH	OH	H	OGlu	H	H	OH	OCH_3_	OH	H
**42**	Chrysosplenetin (5,4’-Dihydroxy-3,6,7,3’-tetramethoxy flavone or Quercetagetin-3,6-7,3’-tetramethyl ether)	OCH_3_	OH	OCH_3_	OCH_3_	H	H	OCH_3_	OH	H	H
**43**	3,5-Dihydroxy-3’,4’,6,7,-Tetramethoxyflavone	OH	OH	OCH_3_	OCH_3_	H	H	OCH_3_	OCH_3_	H	H
**44**	Syringetin (Myricetin-3’,5’-dimethyl ether)	OH	OH	H	OH	H	H	OCH_3_	OH	OCH_3_	H
**45**	Isokaempferide (5,7,4’-Trihydroxy-3-methoxyflavone or Kaemferol-3-methyl ether)	OCH_3_	OH	H	OH	H	H	H	OH	H	H
**46**	Quercetagetin 3,4’-dimethyl ether	OCH_3_	OH	OH	OH	H	H	OH	OCH_3_	H	H

## 4. *A. annua* Has Different Chemotypes

*Artemisia annua* (Asteraceae) is one of over 300 species of the genus *Artemisia* and the major source of artemisinin, although artemisinin has also been found in small concentrations in *A. apiacea* [30] and *A. lancea * [31]. *Artemisia annua* is a C-3 plant more adapted to temperate climates [32] similar to its probable origins in the Sichuan Province of China, but currently-developed cultivars have adapted very well to several countries in Asia, Africa, North and South America and grow in a variety of soils and latitudes varying from about 40⁰ N to 40⁰ S, although it has not adapted well to latitudes lower than 5 degrees from each side of the Equator, growing better in high altitudes where the latitude is so low [33]. The whole family is recognized as a rich source of terpenes and artemisinin is certainly a distinguished example of an important sesquiterpene found in *A. annua*, but the genus is also a rich source of hydroxylated and methoxylated flavonoids.

Although there are many flavonoids that have been isolated from *A. annua*, we will focus on flavonoids tested for antimalarial, anti-cancer, antioxidant or other relevant biological activity *in vitro* and *in vivo*. In field cultivation of a *A. annua* cultivar of Italian origin, eupatin, chrysoplenetin, casticin, and artemetin were the main flavonoids identified [34]. Some of these compounds (e.g., casticin and chrysoplenetin), and artemisinin (0.22 g/100 g), reached the highest levels in flowers and leaves during full bloom. Although the peak artemisinin at full flowering for this low-artemisinin Italian cultivar agrees with others [35] who cultivated a low-artemisinin Chinese cultivar, the high-artemisinin cultivars currently grown in Africa, China and Brazil reach their artemisinin peak before flowering and the seasonal accumulation of these flavonoids might also be different. However, if the high concentrations of polymethoxylated flavonoids are reached during flowering stages and assuming those are of better therapeutic value than the hydroxylated flavonoids, investigation on their anticancer and antimalarial activity, and on their synergistic effects with other compounds should include extracts made of flowering plant material and not only vegetative material, where artemisinin is produced at higher concentrations [36]. Thus, accepting the fact that *A. annua* has different chemotypes [37], different genetic backgrounds might lead to differences in total flavonoids and flavonoid accumulation. Recently, a Brazilian cultivar bred in Campinas, Brazil, was found to contain more flavonoids than a Chinese commercial cultivar [38]. The same Brazilian cultivar was also found to have higher ORAC values than a Chinese cultivar, indicating a higher content of antioxidant components, probably flavonoids [39]. Besides flavonoids, artemisinin and its precursors also vary in *A. annua* with some plants reported to be higher in artemisinic acid (1.9% w/w) than in artemisinin (0.16%) [40], which is opposite than what occurs in commercial cultivars used in Asia, Africa, and Brazil. This occurrence of different chemotypes with high artemisinin and dihydroartemisinic acid, but low artemisinic acid or with low artemisinin and dihydroartemisinic acid, but high artemisinic acid has been previously reported [41]. Thus, for any study using extracts, an HPLC analysis providing a profile and the quantification of the target metabolites is minimally required to allow to interpret and compare the results between different experiments.

## 5. Antioxidant *vs.* Biological Activity of Flavonoids

Antioxidant compounds can delay or inhibit oxidation of lipids and other molecules by inhibiting the initiation or propagation of oxidizing chain reactions. Flavonoids are well known for their antioxidant capacity due to their redox properties, and it has been assumed that a diet rich in flavonoids is inversely correlated with cell aging, lipid peroxidation, cancer, *etc.* However, it is not well established how flavonoids exert their beneficial action and it seems that the best antioxidant flavonoids, such as quercetin, are not necessarily the ones with the best bioavailability, stability, and biologic effect. While an inverse association between the development of lung cancer and the consumption of the highly-hydroxylated quercetin (from onion and apples) and naringin (from white grapefruit) by humans was found [42], methoxylated flavones have more recently been cited to be more stable and present in much higher amounts than their hydroxylated counterparts in pharmacokinetic studies done in rats using chrysin and its methoxylated version 5,7-dimethoxyflavone [43]. Furthermore, most dietary flavonoids from food sources are glycosylated, thereby further decreasing their bioavailability. Thus, it may well be that the inherent antioxidant activity of flavonoids and other polyphenols, which was for a long time believed to be directly correlated with their health effects, has nothing to do with their final biological effects. 

More and more evidence is available that the protective effect of flavonoids against oxidative stress is not mediated by direct radical scavenging. For example, it was shown that relatively low concentrations of flavonoids can increase intracellular glutathione levels via induction of transcription of gamma-glutamylcysteine synthetase [44]. Interestingly, this activity was selective for quercetin, kaempferol, and apigenin, whereas the more potent antioxidant flavonoid myricetin failed to induce the transcriptional activation. Thus, it may well be that the prooxidant properties of some flavonoids may be instrumental for their final health beneficial properties, via interaction with transcriptional activities modulated by antioxidant-response/electrophile-response elements with the Nrf-2-KeaP-1 system as a major target [45]. Repeated mild cellular oxidative stress induced by flavonoids may boost cellular antioxidant defense systems and on the long term may shift the defense system to a higher steady state, thereby preventing disease development or reducing the impact of oxidative stress when disease occurs [46].

Furthermore, many flavonoids are extensively metabolized upon absorption (mostly phase II). It has been shown that flavonoid sulphates and glucuronides may at least in part be responsible for the beneficial effects of the oral intake of flavonoids. It was found that physiological levels of quercetin-3’-*O*-sulphate and quercetin-3-*O*-glucuronide (1 µM), the main circulating metabolites after consumption of quercetin-*O-*glucoside-rich diets by humans, effectively prevented peroxynitrite-induced nitrotyrosine formation in human serum albumin in *in vitro* experiments [47]. Interestingly, only quercetin-3’-*O-*sulphate and quercetin itself was found to inhibit receptor-mediated contractions of the porcine isolated coronary artery by an endothelium-independent action, whereas quercetin-3-*O-*glucuronide was inactive [48]. Such results add to our further understanding of the complexity of the biological activities exerted by flavonoids and their metabolites as a more plausible explanation in comparison to the mere simple direct anti-oxidant activity. Thus, although measuring antioxidant activities of natural products *in vitro* or by methods based on chemical quenching of standard oxidants (ORAC, FRAP, *etc.*) is still valuable as quick reference methods for the presence of polyphenols, *in vivo* tests involving pharmacokinetics or *ex vivo* tests involving animal model systems closely related to humans (such as the porcine model) must be used to understand the final biological response.

## 6. Antioxidant Activity of *A. annua* Flavonoids

*Artemisia annua* contains hydroxylated flavonoids that can be extracted in polar media, such as water or hydro-alcoholic solvents, but also has polymethoxylated flavonoids such as chrysosplenol D, eupatin, cirsilineol, casticin, chrysoplenetin, and artemetin that are more efficiently extracted with low polarity solvents such as dichloromethane [38]. It is obvious that the role of flavonoids, be it direct or indirect, in disease prevention and amelioration is far from resolved. However, there is plenty of evidence that shows the beneficial effects and inverse relationships with cardiovascular disease, cancer, and also with parasitic diseases such as malaria. Evidence has been also accumulating on the synergistic effects of flavonoids with anticancer (Table 1) drugs and with anti-parasitic drugs, besides the facts that some flavonoids have curative effect on their own (discussed further).

As we will approach the traditional artemisia tea later on, one might ask what would be the antioxidant activity of a water extract considering that the oxygen radical absorbance (ORAC) capacity test is performed in dry plant material extracted with 70% acetone, not the traditional way to make tea. To that effect, the screening of 45 Chinese plants for their antioxidant activity was performed by a modified ORAC in which the extraction was done with water at room temperature by vortexing for 2 hours and sonication for 15 minutes, instead of with 70% acetone [49]. The authors lyophilized the water extracts by vacuum and then performed the ORAC test. Two species of *Artemisia* took the 4th and 10th places for the highest ORAC values (µmoles of Trolox equivalents/g). Those were *A. anomala* (ORAC = 1400) and *A. argyi* (ORAC = 1150). Another report indicated that boiling *A. annua* for 1.5 hour in water followed by cooling and lyophilization also resulted in high ORAC values (2123 µmole∙TE/g) while 70% ethanol extracts of the same plant material resulted in 2535 µmoles TE/g [39]. This indicates that the antioxidant capacity of *A. annua* is stable to boiling, but there is no information on what type of flavonoids and phenolic acids are present in these water extracts. However, we assume that boiling water, but not water at room temperature, will extract some of the methoxylated flavonoids detected in dichloromethane extracts mentioned above. We also know that artemisinin, although mostly destroyed by boiling, will be present in at least 70% of the concentration found in hexane extracts when the plant material is steeped in hot water at 85–90 °C [50]. 

## 7. Antimalarial Activity of Flavonoids

Our literature review reveals that some flavonoid derivatives show antimalarial activity. Dehydrosilibin and dimethylallyl campferide were active against several strains of chloroquine-resistant *Plasmodium falciparum in vitro *with IC_50_ values ranging from 1.7 to 24 µM, equivalent to 0.8 to 11.5 µg/mL [51]. However, the naturally-occurring flavonoid silybin had no antiplasmodial activity. The mode of action of sylibin derivatives is unknown and although they had no synergistic effect with chloroquine, they reversed chloroquine resistance or affected *Plasmodium* p-glycoproteins. These authors also reported the effect of the silybin derivative dehydrosilybin on p-glycoprotein-like transporters in the protozoa *Leishmania* spp., leading to the parasite sensitization to daunomycin. Unfortunately, not all is good news for flavonoids and malaria. Rutin was reported as inactive against avian malaria caused by artificial infection with *Plasmodium* (*Bennettinia*) *juxtanucleare* and actually caused a three-fold increase in mean parasitemia (over a 30 day period) compared to the untreated control [52]. *Bennettinia* is a sub-genus of the genus *Plasmodium* that infects avian hosts. However, chloroquine only reduced parasitemia in those chickens in 56.6%. Unfortunately, this study only tested rutin while other flavonoids could result in better anti-plasmodial activity. 

Eleven purified flavonoids from dietary sources were tested *in vitro* for their antimalarial activity against a chloroquine-sensitive (3D7) and a chloroquine-resistant (7G8) strain of *P. falciparum*. Eight showed activity against the 3D7 strain, with IC_50_ values ranging from 11–66 µM, but all showed measurable activity against the chloroquine-resistant 7G8, with activities ranging from 12 to 76 µM [53]. Both 3D7 and 7G8 were most affected by luteolin (at 11 and 12 µM, respectively) and quercetin (at 15 and 14 µM, respectively), followed by apigenin (at 20 and 13 µM, respectively). An exception was noted for acacetin, which was fairly active against the chloroquine-resistant 7G8 strain (IC_50_ = 13 µM), but had much lower activity (IC_50_ > 100 µM) against the chloroquine susceptible 3D7 strain. When the most active flavonoids or all 11 flavonoids were combined, there was an additive effect of the flavonoids. This indicates that a flavonoid-rich (plant-based) diet can play an important role in highly endemic malaria regions. It is also known that morbidity caused by malaria is higher in children under the age of five and pregnant women which are either malnourished or have compromised immune systems (or both). Thus, the sole fact of providing proper nutrition and increasing antioxidant levels through diet would have a pharmacological value to these afflicted populations. In addition, because antioxidant flavonoids are associated with immune systems modulation, children receiving a healthy level of flavonoids in their diet could have a better chance to cope with and overcome *P. falciparum* infections.

## 8. Antimalarial Activity of *A. annua* Flavonoids

Several flavonoids have been extracted and reported from *A. annua* (Table 1). Some flavonoids from *A. annua* and their *in vitro * anti-plasmodial activity were recently cited [54] as the possible synergistic compounds found in the antimalarial tea, although their IC_50_ were much higher than that of artemisinin (0.03 µmol/L). These flavonoids were the following (with IC_50_ against *Plasmodium* in µmol/L): Artemetin (26), casticin (24), chrysoplenetin (23), chrysosplenol-D (32), cirsilineol (36), and eupatorin (65), although it is improbable that these high concentrations used *in vitro* could be replicated *in vivo* after oral delivery of these flavonoids. For instance, humans fed onions as the source of quercetin had an average blood quercetin level of 196 ng/mL after 2.9 h of food intake with levels going down to 10 ng/mL after 48 h [55].

Chrysosplenol-D, quercetin-glucoside, flaviolin, rhamnetin, and pilloin were also reported as major leaf flavonoids of *A. annua* [25]. Also, methoxylated flavonoids have been linked to artemisinin activation *in vitro*, maybe facilitating artemisinin interaction with heme [56] leading to the release of the artemisinin peroxide that is responsible for its antimalarial effects.

Casticin and artemetin had no antiplasmodial activity *in vitro* when tested alone from 10^-9^ to 10^-7^ M, but showed synergistic effects when combined with artemisinin, both at 5 µM [28]. These authors noted that casticin had a better synergistic effect than artemetin, but neither had remarkable synergistic effect with chloroquine. Casticin is similar to artemetin, but has OH groups in A(R_5_) and B(R_4’_), while artemetin has an OH group on A(R_5_), but OCH3 groups in A(R_6_), A(R_7_), C(R_3_), B(R_4’_), and B(R_5’_) (Table 1). The antiplasmodial activity of flavonoids from *A. annua* was confirmed *in vitro* by evaluating their inhibition of the incorporation of hypoxanthine by *Plasmodium* [57]. Artemetin, casticin, chrysosplenetin, chrysosplenol-D, circilineol and eupatorin all had no antiplasmodial activities on their own at 5.0 µM, but had antiplasmodial activity when tested at concentrations ranging from 23–65 µM or higher. Interestingly, these flavonoids except eupatorin, potentiated the antiplasmodial activity of artemisinin at 10^-8^ M when used at 5µM. Recently, quercetin at 1.0 mM was shown to have *in vitro* activity against *P. falciparum* strain 3D7 and had synergistic activity when combined, at 1.0 mM, with artemisinin at concentrations ranging from 0.626 to 20nM. It remains to be seen if the blood concentrations of quercetin obtained after oral consumption of dietary sources, as stated previously, could achieve a similar effect. At the highest artemisinin concentration tested (40 nM) the combined effect of quercetin and artemisinin was similar to the effect of artemisinin alone [58]. Quercetin was shown to inhibit mammalian thioredoxin reductase and the inhibition was dependent on the redox environment [59]. That enzyme was also shown to be essential for the survival of the erythrocytic stage of *P. falciparum* [60] probably accounting for the synergistic effect of quercetin with artemisinin on *P. falciparum*, which might possess a thioredoxin reductase enzyme form that is more affected by quercetin than the mammalian counterpart. The methoxylated flavonoids artemetin, chrysoplenetin, chrysosplenol-D, and circilineol were all present in chloroform extracts of *A. annua* and were linked to the *in vitro* anti-plasmodial activity of either whole plant or cell cultures [57]. 

Considering that *Plasmodium *spp*.*, besides depending on cyclin-dependent kinases, also have a putative serine-threonine kinase with considerable homology to other serine-threonine kinases [61] that hold affinity to flavonoids, it would be logical to expect that flavonoids that inhibit these kinases could also exert the same effect on *Plasmodium* kinases, thus hindering the protozoa development or proliferation in the body.

## 9. Flavonoids and Cancer

Unlike malaria, it is generally accepted that flavonoids prevent, delay, or help cure cancer. Thus, several reviews associating flavonoids from beverages (such as tea and wine), fruits, vegetables, and herbals with cancer can be found [62,63,64]. Flavonoids can affect cancer in different, but not always clear ways. Flavonoids can prevent DNA mutations that occur in critical genes, such as oncogenes or tumor-suppressing genes, thus preventing cancer initiation or progression [65]. These authors have cited studies where flavonoids, as antioxidants, inhibited carcinogenesis, and that some flavonoids such as fisetin, apigenin, and luteolin (the latter two produced by *A. annua*) are potent inhibitors of cell proliferation, while others inhibit angiogenesis. An inverse correlation has been found between dietary flavonoid intake and subsequent lung cancer occurrence [62], and quercetin (wide spread in plants, including *A. annua*) intake from onions and apples was inversely associated with the risk of having lung cancer in a study involving 9,959 Finish men and women aged 15–99, with onions being effective particularly against squamous-cell carcinoma [42]. A current review mentions the involvement of flavonoids with the modulation of p-glycoproteins, the proteins associated with multi-drug resistance, inhibition of several enzymes of the CYP450 family, and modulation of phase-II enzymes. Because both classes of enzymes are involved in drug metabolism and in the process of chemical carcinogenesis, interaction of flavonoids with these enzyme systems hold great therapeutic potential for detoxification, chemoprevention and the suppression of drug resistance [62]. Although there are hundreds of flavonoids that can act as modulators of oxidative stress, anti-cancer, and as drug synergists by inhibiting the enzyme systems mentioned above, we will focus on the main flavonoids produced by *A. annua* and that have been cited for their antioxidant and anti-cancer activity.

## 10. Anticancer Activity of Artemisinin and *A. annua* Flavonoids

Artemisinins show promising anti-cancer activities when tested *in vitro* and *in vivo* [66,67,68,69]. Artemisinins contain an endoperoxide group that is essential for their antimalarial and anticancer activities. Like hydrogen peroxide, H_2_O_2_, artemisinin reacts with ferrous iron, Fe^2+^, to generate radical species. The short-lived artemisinin-generated radical species have been linked to its anti-parasitic and anti-cancer activities. The anti-cancer activity of artemisinin derivatives can significantly increase when iron complexes are added in the cell culture medium [3,66]. A covalent conjugate of artemisinin and transferrin (ART-Tf), an iron transport protein in human, is actively taken up by cancer cells through the transferrin receptor (TfR)-mediated endocytosis pathway, and shows significantly higher anti-cancer activity than unconjugated artemisinin [70,71]. Like ART-Tf, artemisinin-peptide conjugates that are designed to target TfR also showed highly potent and selective anti-cancer activities [72]. These studies show the importance of iron metabolism in determining the effectiveness of artemisinin derivatives in killing cancer cells. Artemisinin derivatives induce programmed cell death of cancer cells by activating the intrinsic or the cytochrome C-mediated pathway for apoptosis, although the initial protein targets of artemisinin derivatives for apoptosis in human cancer cells have not yet been identified [71]. Although the generation of free radicals originating from the reaction of artemisinin with molecular iron is mentioned as one of the main mechanism for its anticancer activity, there are other mechanisms, crucial for cancer proliferation and survival that are affected by artemisinins. These mechanisms have been described in a current review [73] and will not be discussed here.

**Table 2:** Synergism between flavonoids and anticancer drugs.

Flavonoids	Mean GI_50_ (μM) [74]	Synergy with anti-cancer agents
**Eupatin**	4	mitoxantrone [75]
**Silybin**		Paclitaxel [76], TRAIL [77], SN-38 [78], mitoxantrone [78], cisplatin [79], carboplatin [79]
**Quercetin**	60	TRAIL [80], cisplatin [81], doxorubicin [82], vinblastine [83], paclitaxel [83], gemcitabine [84], topotecan [84]
**Apigenin**	27^a^	TRAIL [85,86], tamoxifen [87], fulvestrant [87]
**Luteolin**	N/A	Rapamycin [88], doxorubicin [89], cisplatin [90], TRAIL [91]
**Kaempferol**	N/A	TRAIL [92,93], vinblastine [83], paclitaxel [83], mitoxantrone [78]

^a^ The value was taken from the NCI website, http://dtp.nci.nih.gov/docs/dtp_search.html.

The synergism of flavonoids and artemisinin in cancer treatment has not been reported. Flavonoids can potentially enhance the anti-cancer effect of artemisinins by increasing their bioavailability and serum half-life values [66], inhibiting metabolic enzymes [67], increasing the cellular Fe^2+^ level by reduction of Fe^3+^ [68], and by affecting key pro-apoptotic and anti-apoptotic proteins in cancer cells. The following sections summarize the anti-cancer activity of flavonoids found in *A. annua* extracts. Some of them show a potent synergism when combined with anti-cancer drugs (Table 2).

### 10.1. Flavones

Pilloin has shown a weak growth inhibition effect on transformed lymphocytes at a concentration of 3.0 mM [94]. Apigenin, besides *A. annua*, is widely found in edible plants such as celery, parsley, thyme, red pepper, onion, lettuce, berries, *etc*., and has been extensively studied for its anti-cancer effect [95]. When tested on A2780 (human ovarian cancer) cells, apigenin arrested cells at G2/M mitotic phase, and induced apoptosis at the concentration of 40 μM [96]. Daily i.p. (intraperitoneal) injection of apigenin to a mouse xenograft model with subcutaneous implantation of A2780 cells significantly reduced tumor mass compared to the control group. Western blot analyses showed that apigenin suppressed the expression of ld1 (inhibitor of differentiation or DNA binding protein through activation transcription factor 3 or ATF3). Apigenin was also recently tested on S2-013 and CD18 (human pancreatic cancer) cell lines [97]. In the study, apigenin was shown to inhibit the expression of GLUT-1 glucose transporter at a concentration of 25 μM. Glucose transporters are generally over-expressed in cancer tissues, and its over-expression is a poor prognosis factor in colorectal, breast, ovarian and gastric carcinomas. Apigenin also inhibited the phosphoinositol 3-kinase (PI3K)/Akt pathway, inducing the growth inhibition of the pancreatic cancer cells.

Luteolin is widely distributed in many types of plants including fruits, vegetables, and medicinal herbs. Its anti-cancer activity has been reviewed extensively [98,99]. Luteolin induces apoptosis and inhibits cell proliferation, metastasis and angiogenesis. When tested *in vitro*, IC_50_ values ranged between 3 and 50 mM, and luteolin was active when tested in xenograft cancer models. Luteolin's cytotoxicity appeared to be associated with suppression of PI3K/Akt pathway, nuclear factor kappa B (NF-kB), and X-linked inhibitor of apoptosis protein (XIAP). Luteolin was shown to increase intracellular reactive oxygen species (ROS) in human hepatic cancer cells [100]. Proteomics analyses showed that peroxiredoxin (PRDX6) and prohibin (PHB) are key targets of luteolin. These two proteins are involved in ROS metabolism and apoptosis induction.

Eupatorin was found in various medicinal plants, besides *A. annua*. The compound has shown a moderate cytotoxic effect [101,102] on MK-1 (human gastric adenocarcinoma), HeLa (human uterus carcinoma), B16F10 (murine melanoma), and 26-L5 (murine colon cancer) cell lines. Interestingly, the same compound was totally inactive against P-388 (lymphatic leukemia) cell line [103]. Eupatorin inhibited the growth of MDA-MB-468 (human breast carcinoma) cell line in a dose dependent manner with an IC_50_ of 0.5 μM. In the same assay, eupatorin showed only weak inhibitory effect against MCF-10 (normal mammary tissue) cells. Eupatorin was found to be metabolized to the flavone cirsiliol and two other unidentified metabolites by a CYP450 enzyme, CYP1A1 [104]. The enzyme is present in MDA-MB0468 cells, but not expressed in MCF-10A healthy mammary cells. A similar activation of eupatorin by a CYP450 enzyme has also been observed in MCF-7 (human breast adenocarcinoma) cells [105].

Cirsilineol was tested on three cancer cell lines, HeLa, MK-1 and B16F10 [106]. Although cirsilineol was more potent than eupatorin against MK-1 cells, it was not effective against HeLa cells. Both cirsilineol and eupatorin showed similar activity against B16F10 cells. In a separate study [107], cirsilineol inhibited the growth of four cancer cell lines, Caov-3, Skov-3, HeLa, PC3 and HepG2. Interestingly, cirsilineol had no effect on a normal human liver cell line (L02). Cirsilineol induced apoptosis in Caov-3 cells by releasing cytochrome-c from mitochondria, followed by activation of caspase-9, -3 and PARP proteins.

### 10. 2. Flavonols

Eupatin was tested on the NCI 60-cell line *in vitro* screen, and the mean GI_50_ value was 4 μM [108]. The growth inhibition data of structurally similar flavonoids suggest that anti-mitotic mechanism may be responsible for the anticancer activity of eupatin although eupatin itself is a poor inhibitor of tubulin polymerization. Eupatin was also highly active as a free radical scavenger when tested against the oxidizer 1,1-diphenyl-2-picrylhydrazyl (DPPH) [109]. Eupatin has been found to be a moderately potent inhibitor of ABCG2, breast cancer resistance protein (BCRP) or mitoxantrone resitance protein (MXR) [110]. The IC_50_ value of eupatin for ABCG2 inhibition was 2.2 μM against (ABCG2 or BCRP). ABCG2 appears to be involved in the resistance to several chemotherapeutic agents, and eupatin and its derivatives may be a useful synergistic flavonoid in reversing the drug resistance of cancer cells.

Quercetin is widely distributed in the plant kingdom, and has been extensively studied for its biological activities including anticancer activity. Quercetin inhibited the growth of MCF-7 breast cancer cell line with an IC_50_ value of 5.2 mg/mL [111]. Quercetin has been shown to inhibit protein kinase C and to bind to type-II estrogen binding sites. Interestingly, structurally similar flavonoids, myricetyn and epicatechin, did not show significant inhibitory activity against MCF-7 [112], indicating that some specific structure-related interactions between quercetin and its potential cellular target(s). Epicatechin, unlike quercetin, lacks an acetal group on C4 position. Quercetin also inhibits the growth of HT-29 colon cancer cell line with an IC_50_ = 81.2 mM [113]. In the presence of 150 mM of quercetin, surface expression of alkaline phosphatase was increased along with a marked increase in caspase-3 activity that lead to apoptosis. Quercetin synergized with cisplatin when tested against OVCA433 human ovarian cancer cells [114]. The synergy has been explained by the specific interaction between quercetin and type-II estrogen binding sites. Similar correlation between growth inhibition effect of quercetin and presence of type-II estrogen binding sites has been observed on colon and colorectal cancers. Quercetin also induced apoptosis on HSC-2 squamous cell carcinoma and HL-60 promyelocytic leukemia cells. Western blots analysis did not show any consistent changes in Bax, Bad and Bcl-2 proteins upon quercetin treatment [116].

Quercetagetin 6,7,3’4’ tetramethyl ether (differ from artemetin only by having a OH on C-R_3_) was reported to be extremely effective *in vitro* against tumor cell lines P-388 (murine lumphocytic leukemia), A-549 (human lung carcinoma), MCF-7 (human breast adenocarcinoma), HT-29 (human colon adenocarcinoma), and KB (human nasopharynx carcinoma) with ED_50_ values of 4.9 × 10^-1^, 4.81 × 10^-1^, 2.47, 1.25, and 6.80 × 10^-1^ µg/mL, respectively, while its 3-O-methyl analog artemetin (Table 1, No. 18) was not effective against any of these tumor cells [117]. Quercetagetin anticancer effects were recently confirmed when it was reported as a potent and selective inhibitor (IC_50_ = 0.34 µM) of a serine-threonine kinase (PIM1), implicated in the development of leukemia, lymphoma, and prostate cancer [118]. In the same study, apigenin, quercetin, kaempferol, and luteolin (all found in *A. annua* and other plants) also inhibited PIM1 with IC_50_ of 0.94, 1.1, 1.3, and 1.6 µM, respectively. The authors presented evidence that quercetagetin is a direct ligand for the ATP-binding pocket of PIM1 kinase. However, it is known that kinase inhibitors usually bind to ATP-binding sites as reported for cyclin-dependent kinases involved in diseases such as cancer, viral infections, and infection by protozoan agents such as *Plasmodium* sp. and *Leishmania* [119]. Afinity to ATP-binding sites is a characteristic of several kinase inhibitors, including the flavonoid flavopiridol (undergoing clinical trials for cancer treatment) and quercetagetin [118]. 

Chrysosplenetin has been tested for its anti-cancer activity on Lu1, LNCaP, MCF-7, and HUVEC cell lines, and has been found to be inactive (ED_50_ > 5.0 μg/mL) [120]. Although one study cannot rule out the possible beneficial effect of chrysosplenetin, it has a striking structural similarity with quercetin, which is active against cancer, inhibits protein kinases, *etc*., as previously described but, unlike quercetin, the positions C3, A7, and B3’ are occupied by CH_3_ groups in chrysosplenetin, what might make it less active than quercetin. Another factor to consider is the threshold established (in this case 5.0 µg/mL) and used as a guide of activity, or lack of, in both *in vitro* and *in vivo* screening assays.

Casticin has exhibited a potent anticancer activity against PC-12 (GI_50_ = 114 nM) and HCT116 (GI_50_ = 119 nM) [121]. The activity was comparable to that of cisplatin. Casticin was also tested on two human epidermoid carcinoma cell lines, KB and A431 [122]. While the growth of KB cells was significantly inhibited by casticin (IC_50_ = 0.23 μM), the compound had only a minimal effect on the growth of A431 cells. Two normal cell lines, 3T3 Swiss Albino (mouse) and TIG-103, were not affected by casticin. Flow cytometry analyses showed that casticin induced significant arrest of KB cells at the mitotic phase G2-M. Casticin disrupts mitotic spindles that may be responsible for the G2-M arrest of KB cells. Similar observations have been reported with MCF-7 breast cancer sublines MN1 and MDD2 [123]. Casticin treatment increased the expression of p21, resulting in the inhibition of cyclin-dependent kinases (Cdk). Furthermore, casticin inhibited the expression of cyclin-A and Bcl-2 proteins, inducing apoptosis of these cells. It has also been suggested that casticin inhibits *anticancer*, and may be able to reverse drug resistance when combined with other anticancer drugs, including artemisinin which seems not to exert any inhibition on *anticancer* by itself [124].

Artemetin is structurally very similar to casticin, which differs only by a hydroxyl group on the 3' position of the B ring, occupied by a methyl group in artemetin. Artemetin showed a moderate anti-cancer activity (IC_50_ = 16 μM) when tested on two human epidermoid carcinoma cell lines, KB and A431 [122]. The activity was 7–8 times lower than that of casticin. Artemetin has shown a significant anti-cancer activity against HL60 (human promyelocytic leukemia) cells with an IC_50_ = 6.44 μM [125].

Chrysosplenol-D inhibited the growth of KB cells with an ED_50_ = 13.95 μg/mL [126]. In a study with HeLa cells, chrysosplenol-D markedly inhibited the incorporation of 32P into phospholipids when the cells are stimulated by 12-O-tetradecanoylphobol-13-acetate (TPA) [127]. Chrysosplenol-D is one of the active ingredients in Fructus Viticis (*Vitex trifolia*), a traditional Chinese medicine, that has been used to treat human cancer. Chrysosplenol-D inhibited the growth of tsFT210 (a mouse cdc2 mutant) cells with an IC_50_ = 3.5 μg/mL by inducing apoptosis.

Rhamnetin inhibited the growth of HeLa cells with an IC_50_ = 7.0 μg/mL [128]. Rhamnetin and other flavonoids were evaluated for their ability to inhibit cyclooxygenase-2 (COX-2). COX-2 plays an important role in cancer development. Both quercetin and rhamnetin inhibited the growth of DLD-1 (human colon adenocarcinoma) cell line with a remarkable suppression of COX-2 transcriptional activity (LD_50_ = 18.6 mM for rhamnetin) [129]. Rhamnetin also showed a moderate antiproliferative activity against MDA-MB-435 (ER- human breast cancer), MCF-7, DU-145 (androgen receptor negative prostate cancer), HT-29 (human colon cancer), DMS-114 (human lung cancer), and SK-MEL5 (human melanoma) cell lines with IC_50_ values of 22 - 85 μM [130]. Rhamnetin and other related flavonoids have been suggested to bind to vascular endothelial growth factor (VEGF), based *in silico* screening [131].

Kaempferol, in addition to *A. annua*, has been isolated from *Ginkgo biloba*, a popular medicinal plant in Asian countries. Kaempferol inhibited the growth of ovarian cancer cell lines, OVCAR-3 and A2780/CP70 [132]. The effect was moderate, 91% inhibition of OVCAR-3 cells and 94% inhibition of A2780/CP70 cells by 20 μM and 40 μM kaemferol, respectively. Interestingly, kaempferol significantly reduced both angiogenesis and VEGF gene expression at both mRNA (transcriptional) and protein (translational) levels. Both hypoxia inducible factor-1α (HIF-1α) and ESRRA were also down-regulated by kaempferol. Both proteins are involved in regulating VEGF expression. Kampferol induces apoptosis in MCF-7 cells at a concentration of 50 μM [133]. Western blot analyses show the cleavage of PARP and activation of caspases-7 and 9 as well as an increase in Bax expression. In a separate study with multidrug resistant cells, MCF-7/ADR, kaempferol was shown to inhibit p-glycoprotein with an efficacy similar to that of verapamil [134]. The growth of another breast cancer cell line, MDA-MB-453, was also inhibited by kaempferol [135]. When the cells were exposed to kaempferol, cell cycle arrest occurred at G2/M phase, apparently caused by the down-regulation of CDK1 and cyclin A and B. Kaempferol and other two flavonoids, quercetin and myricetin, prevented the cell migration and metastasis of DAOY (medulloblastoma) cells by inhibiting hepatocyte growth factor (HGF)/Met signaling [136]. Among the three flavonoids tested, kaempferol was the most effective with an IC_50_ value of 0.5 μM. Kaempferol was ineffective in inhibiting the growth of human glioma cells U251 and U87. However, exposure to kaempferol sensitized these cell lines to tumor necrosis factor-related apoptosis ligand (TRAIL) [92,93]. Kaempferol induced the proteosomal degradation of survivin, thus increasing the sensitivity of the treated cells to TRAIL-induced apoptosis. Kaempferol was also recently shown to be moderately-active against pancreatic cancer [137], prostate cancer [138], and lung non-small cell carcinoma [139] *in vitro*.

Isorhamnetin is not only found in various plants, but also is an immediate metabolite of quercetin in mammals. Isorhamnetin inhibited the growth of BEL-7402 (human hepatocellular carcinoma) cells with an IC_50_ = 74.4 μg/mL at 72 h [140]. Incubation with 50 mg/mL of isorhamnetin induced apoptosis in 13.77% of BEL-7402 cells. The growth of Eca-109 (human esophageal squamous carcinoma) cells was inhibited by isorhamnetin, with an IC_50_ = 40 μg/mL [141]. Western blot analyses showed that incubation with isorhamnetin decreased the expression of Bcl-2, c-myc and H-ras while the expressions of Bax, c-fos and p53 increased. Isorhamnetin induced apoptosis in LLC (Lewis lung cancer) cells, and inhibited the cellular growth with an IC_50_ value of 40 μM [142]. The apoptosis was mediated by the release of cytochrome-c from mitochondria, and subsequent activation of caspase enzymes. Mouse xenograft model with LLC cells showed that i.p. injections of isorhamnetin (0.5 mg/kg/day) significantly reduced the tumor volume. The efficacy was about 10 times better than that of quercetin.

Astragalin is a 3-O-β-D-glucoside of kaemferol. Astragalin was tested on several cancer cell lines, including DU-145 [143], GLC4 (human small cell lung carcinoma), and COLO 320 (human colorectal cancer) [144], with only a weak growth inhibitory effect.

Isoquercitrin and quercimeritrin are the 3-O-β-D-glucoside and 7-O-β-D-glucoside of quercetin, respectively. In an *ex vivo* angiogenesis assay, isoquercitrin had the strongest activity, completely inhibiting microvessel growth at 100 μM [145]. Both quercetin and quercimeritrin had a weaker effect on angiogenesis. Quercetin, isoquercitrin and quercimeritrin inhibited the growth of HUVEC cells at 100 μM, but only quercetin and isoquercitrin were able to inhibit the HUVEC tube formation.

Other flavonoids including flaviolin, luteolin 7-methyl ether, tomentin, isokampferide, luteolin-7-methyl ether, quercetagetin 3-methyl ether, luteolin 7-methyl ether have been isolated from *Artemisia annua* [146], but their anti-cancer activities have not been well studied.

## 11. Flavonoid Metabolism

For the flavonoids to exert their putative role as oxidative stress modulators, antimalarial, anticancer, or synergistic effects, they are required to be absorbed from the gastrointestinal tract. Pig microbiota has been studied by fluorescence *in situ* hybridization and found to be very similar to human microbiota [147]. Phenolic acids have shown good absorption in the gut, leading to their potential antioxidant effects in the blood [148]. Thus, although bioavailability of intact polyphenols might be low, the large pool of phenolics acids resulting from microbial and mammalian metabolism might be responsible for their antioxidant (or stress modulatory) activity [149]. This idea might also hold for flavones present in *A. annua* (Figure 2), believed to have individual or synergistic antimalarial and anticancer activity, but that assumption have not been tested yet. Quercetin and catechin levels peaked at approximately one hour after ingestion by rats with quercetin levels remaining fairly constant in the blood (3–4 µM) for 24 hours. Although a small portion of both catechin and quercetin were eliminated through the feces from 12 to 24 hours after intake, a significant part was absorbed either intact or as metabolites and was detected in the blood, small and large intestine, cecum and to a minor extent in liver and kidney (mostly quercetin), with part being eliminated through the urine [150].

In general, plasma concentrations have been reported to vary from 0.3–0.75 µmol/L after consumption of 800–100 mg of quercetin equivalents present in apples, onions, or meals rich in plant products but values can be as high as 6.0 µmoles/L for naringenin, present in grapefruit juice, after ingestion of 200 mg [151]. While this review does not focus on flavonoid metabolism, its understanding is crucial for designing studies involving these polyphenols in humans and animals. Thus, we recommend the reading of two excellent reviews on bioavailability and bioefficacy of polyphenols in humans [152,153].

## 12. Artemisinin Metabolism and Its Synergism with Synthetic and Natural Products

Metabolic and pharmacokinetic studies of artemisinin derivatives such as artesunate, artemether, and arteether show that they have a short half-life (1–2 h) in the blood when taken orally but have a longer half-life (7–9 h) when taken intramuscularly. *In vitro* metabolic studies with human liver microsomes that had the capability of metabolizing different drugs showed that artemisinin was metabolized majorly by the CYP450 enzyme CYP2B6 with a secondary contribution of CYP34A in individuals with low CYP2B6 expression [154]. Bioavailability of artemisinin was reduced five-fold after five days of continuous oral administration, but p-glycoproteins were not involved in artemisinin clearance from the cells [124].

Malaria infection itself has a significant effect on the pharmacokinetics of artemisinin derivatives. For example, when artesunate was given orally, peak plasma concentrations and the relative bioavailability of dihydroartemisinin (the major metabolite of artemisinin derivatives) were higher in malaria patients given artesunate than in healthy patients [155]. Pharmacokinetic parameters also changed significantly for artesunate and dihydroartemisinin in rats infected with *Fasciola hepatica* compared to healthy animals [156]. Thus, the bioavailability of artemisinin from tea or extracts in humans infected with malaria still needs to be investigated beyond the typical 5–7 days of treatment. The data should be compared to that of pure artemisinin or its derivatives given to healthy volunteers. We speculate that artemisinin (from tea or ACT) will have a better effect if taken for a short period because, after 5–7 days of treatment, pharmacological levels of artemisinin in the blood would decrease significantly due to degradation by induced CYP450 enzymes. Bioavailability of artemisinin derivatives may also change under other disease conditions such as cancer or malaria. For cancer treatment, it would require a longer treatment than that for malaria, and it would be useful to test if simultaneous use of flavonoids would keep artemisinin therapeutic levels in the blood for longer.

Although synergism in some cases can lead to drug overdose, its beneficial aspects can be explored to improve the bioavailability of a drug with a wide safety margin such as artemisinin. In Table 2, we have shown the synergistic effect of flavonoids with anticancer drugs. Here we will report what we found regarding synergy between flavonoids, curcuminoids, and other natural compounds with artemisinin and its related compounds used to treat malaria and other parasitic diseases.

Curcumin fed orally at 100 mg/kg to mice in combination with artemether at 0.75 and 1.5 mg/mouse resulted in better survival rates for mice infected with *Plasmodium berghei* [157]. Although the dose of curcumin used was fairly high, those authors reported that curcumin toxicity is very low and as much as 8.0 g/day was well tolerated by cancer patients for three months without toxicity. Quercetin is wide spread in the plant kingdom and a major flavonoid of *A. annua * [158]. Quercetin was reported to increase the bioavailability of moxidectin (and anthelmintic drug) in lambs [159]. The methoxylated flavonols chrysosplenol-D and chrysoplenetin, which alone had very weak growth inhibitory action, had a synergistic effect with berberine against *Staphylococcus aureus* [160]. The authors attributed this synergism to the inhibition of an *S. aureus* multidrug resistance (MDR) pump. Chrysosplenol-D, chrysoplenetin and other methoxylated flavonols produced by cell cultures of *A. annua* were previously reported to potentiate the activity of artemisinin against *Plasmodium falciparum* [57]. Epigallocatechin gallate and catechin gallate were the most effective catechins from a crude green tea extract to have antimalarial effects of their own and to potentiate the effects of artemisinin *in vitro* [161]. Ellagic acid, found in plants that contain hydrolyzable tannins, was found to have antimalarial effects *in vitro* and *in vivo* (in mice) against *Plasmodium vinckei petteri*. However, although its *in vivo* effects showed 100% parasite growth inhibition when given intraperitoneously at 1.0 mg/kg/day, there was no effect when ellagic acid was given orally, even at 1.0 g/kg/day. This indicates that its oral bioavailability is very poor. The authors reported the synergistic effects (*in vitro*) of ellagic acid with chloroquine, mefloquine, artesunate, and atovaquone, but its slight antagonistic effect with artemisinin [162].

*Artemisia annua* tea was also effective against *Toxoplasma gondii*, although artemisinin was only present at 0.2% in the tea [163]. However, neither the tea nor sulfadiazines were able to control completely the infection with survival ranges from 20–50%. In addition, these authors reported an *in vitro* immunostimulatory activity of the tea. 

Grapefruit juice significantly increased the oral bioavailability of artemether without an effect on the elimination half-life [164]. The authors suggested a possible role of intestinal CYP3A4 in the presystemic metabolism of artemether. In this study, the inhibitory effects of grapefruit juice on CYP450 and CYP2B5 were apparent but serum liver enzymes were unchanged compared with the uninfected control group. Co-administration of grapefruit juice with artemether (150 mg/kg) achieved complete protection of mice from damage induced by *Schistosoma mansoni* infection, eliminated eggs, and prevented pathological granulomatous lesions [165]. According to a recent review [166], the major constituents in grapefruit juice are flavonoids, of which the most prevalent is naringin, which is responsible for the bitter taste of grapefruit and is present at a concentration of up to 1,200 mg/mL. Other flavonoids, such as quercetin and kaempferol, are present in trace amounts. Naringin in grapefruit juice is metabolized *in vivo*, probably in the small intestine, to give the aglycone, naringenin. Furanocoumarins are minor components present in grapefruit juice, and include 6’,7’-dihydroxy-bergamottin and its more lipophilic analog, bergamottin, which is found at the higher concentration of 10 mg/mL. The flavonoids and furanocoumarins of grapefruit juice have the ability to modulate CYP3A4 and p-glycoproteins, raising the potential of drug interactions and thus are considered to have synergistic affects with artemisinin. As the duration of effect of grapefruit juice can last 24 h, repeated consumption can lead to a cumulative effect on the pharmacokinetics of co-administered drugs. Other herbs or herbal compounds that are known to inhibit CYP3A4 (intestinal) are bitter orange, berberine, and piperine [167].

The above examples show that more research should be done on the favorable interactions between artemisinin and the natural antioxidant compounds like the ones cited above. For instance, although these examples provided focus mostly on the antioxidant flavonoids, some diversification towards other metal-chelating, antioxidant, compounds such as the hydrolyzable tannins found in grape seeds, pomegranates, cinnamon, and other natural food sources is necessary. The recent results obtained *in vitro* and *in vivo* on the antimalarial properties of ellagic acid, a major component of hydrolyzable tannins illustrate the need for this diversification [168].

Although examples of natural compounds interaction with artemisinin-derived drugs are not many, there are previous examples in the literature on the synergism between artemisinin and pharmaceutical-grade drugs (some of natural origin) against chloroquine-resistant and -sensitive strains of *Plasmodium falciparum in vitro* such as mefloquine and quinine [169] methylene blue [170]; atovaquone and mefloquine [162], *etc.* Other drugs such as ketokonazole, orphenandrine, and 8-methoxypsoralen inhibited artemisinin metabolism by CYP2B6 enzymes in 46%, 76%, and 82%, respectively [154]. These authors also determined that the latter two drugs, when combined, inhibited artemisinin metabolism by 90%. These results indicate that co-administration of artemisinin with compounds that inhibit CYP2B6 and CYP3A4 might provide a way to keep artemisinin at pharmacologically-active levels in the blood. Some natural dietary compounds have been found to play that role. These synergistic interactions eventually led to the recommendation of artemisinin combination therapies (ACT) to substitute monotherapies based on artemisinin-derived drugs as it has been recommended by the World Health Organization since 2001 and finally agreed upon by pharmaceutical producers in 2006 [171].

The findings reported above indicate that the synergistic interaction of flavonoids and other natural compounds with artemisinin. Thus, the flavonoids, coumarins, and other compounds found in the tea and alcoholic extracts of *A. annua* might increase significantly the efficacy of artemisinin and its derived drugs against parasitic diseases such as malaria. The only pharmacokinetics study available indicates that although absorption of artemisinin from the tea was faster, the bioavailability of artemisinin from tea was similar to the one reported for the pure compound [7]. However, the study was done in healthy volunteers and the whole liter of tea was consumed in five doses of 200 mL in no more than 15 minutes. 

## 13. What Lessons Can We Learn from the Tea?

*Artemisia annua* has been traditionally used for over two thousand years to treat “intermittent fevers” (back then, the word malaria did not exist) and for hemorrhoids. Ge Hong, in the 4th century CE (common era) recommended soaking “a bunch of fresh plant in two sheng (2 × 200 mL) of water, then wringing it out and ingesting the juice in its entirety” [172]. That might have created an emulsion of the water with the essential oils, plant waxes, flavonoids, and quinic acids. However, that author [172] noted that in the history of qing hao (green herb currently identified as *A. annua*) *A. annua* and *A. apiacea* were confused as the same plant. Chinese scholars, even back then, separated the two species as one with markedly blue-green color (blue-green hao, or blue-green herb, or qing hao), which was *A. apiacea*, and another one light-green in color (huang hua hao), which was *A. annua*. Although *A. apiacea* is now known to have a small concentration of artemisinin [30], it has similar flavonoids that might have contributed to its anti-fever properties.

Currently, the recommended tea recipe is made of 4.5–9.0g of dried *A. annua* leaves prepared as an infusion (steeping or immersion in hot water). Although artemisinin is poorly soluble in water, and reported to be present in only 10.6 mg/mL when boiling water is poured over artemisinin [173], hot water (85–90 °C) can extract approximately 80% of the artemisinin extracted by petroleum ether from leaves [6]. In the first clinical trial, approximately 40% of the artemisinin contained in leaves was extracted by adding boiling water to dry leaves, with brief stirring. Boiling the leaves for five minutes reduced the content of artemisinin in the tea [174]. These authors reported a 92% clearance (44 patients) in parasitemia when 48 patients were given the tea four times a day for seven days. Boiling the tea for 1.5 h destroyed approximately 90% of the artemisinin (compared to petroleum ether extracts), but only about 25% of dihydroartemisinic acid and artemisinic acid, as determined by HPLC-UV (Ferreira, unpublished). In addition, the oxygen radical absorbance capacity (ORAC) value of the boiled extract was still almost as high as the antioxidant activity of an ethanolic extract (2,123 *vs.* 2,535 µmoles TE/g) of the same plant material [39]. In a second study using a randomized trial involving 132 patients, the authors [173] determined that the artemisinin content of the leaves was 1.4% and that the tea made with 9.0 g of leaves provided 94.0 mg of artemisinin/patient/day, which is only 19% of the artemisinin provided by a 500 mg/day dose of artemisinin. However, while its known that pure artemisinin availability drops to about 30% by day five of consecutive oral administration due to the action of CYP450 enzymes, it is unknown if the same fact occurs with artemisinin provided with a mixture of flavonoids (as the tea) that are assumed to inhibit CYP450 enzymes. Regardless of blocking CYP450 enzymes or not, both studies resulted in a high recrudescence rate of malaria (also reported to malaria treated by any artemisinin-related drug monotherapy) and the authors did not recommend the tea as a substitute for the currently WHO-recommended ACT. In a study with the tea, artemisinin was absorbed faster from the tea than from artemisinin capsules by human subjects and reached plasma peak concentrations of 240 ng/mL less than an hour from oral intake [7], falling to a little over 50 ng/mL two hours after intake, and approximately 20 ng/mL three hours after intake. According to the authors, the plasma concentration three hours after intake (20 ng/mL) was approximately 40% of the peak concentration reported after oral intake of 500 mg of artemisinin from capsules, but still higher than the 9.0 ng/mL reported to inhibit growth of *P. falciparum in vitro* [54]. Interestingly, the half-life of flavonoids seem to be much longer as reported for quercetin consumed as onions, which peaked in the plasma at 196 ng/mL after 2.9 hours, but that was still present at 10 ng/mL 48 hours after the consumption of the onions [175].

A study using mice infected with *Plasmodium chabaudi chabaudi* compared artemisinin from tea (present at 34 mg/mL) with artemisinin-tea equivalent provided as pure artemisinin (34 mg/mL) and with an artemisinin combination therapy (ACT) at the dose recommended by the WHO [176]. The authors reported that although control mice and mice treated with the pure artemisinin equivalent found in the tea died at day 5, infected mice treated with artemisia tea survived until day 11, and mice treated with the WHO-recommended ACT survived the infection. This shows that although the tea (as currently prepared) is not recommended as a substitute, it could buy valuable time for someone infected with malaria and far from a hospital or clinic. In the Brazilian Amazon or in sub-Saharan Africa, it might take 2–3 days for a person afflicted with malaria to reach a hospital, assuming that the hospital has the proper antimalarial drug in stock, considering the high use of the drug in a highly endemic area.

Most recently, an interesting study published in this special issue compared the traditionally made tea preparation (soaking for 2 and 12 hours and wringing the juice out of the plants) with a more labor intensive (“pounding” fresh plants to produce a thick green extract) and compared these two types of extracts with a 30 mg/kg dose of artemisinin in *Plasmodium berghei*-infected mice [177]. The authors found that, although artemisinin is not soluble in water, soaking fresh branches for 2 and 12 hours did extract artemisinin, although the “pounding” method resulted in about 20 times and four times more artemisinin than the infusion made with the dried branches and the infusion made only by soaking fresh branches for 12 hours, respectively. Interestingly, the authors reported that the “pounded” juice was dark green while the infusions obtained from soaked material were clear. This indicates the presence of chlorophyll extracted from the palisade parenchyma (tissue layer immediately underneath the epidermis) of leaves, where the flavonoids might also be present. The authors reported that the infusions provided from 9–27 mg of artemisinin equivalents/kg of live mouse weight and, compared to the 30 mg/kg of pure artemisinin, the infusions that provided 18 and 27 mg/kg of artemisinin equivalents suppressed parasitemia 2.6 times better than pure artemisinin at 30 mg/kg. Finally, they concluded that artemisinin alone could not account for those results and that only the green pounded juice presented *in vivo* antiplasmodial activity in mice. We see these results as a clear indication that other components extracted in the pounded juice (most probably flavonoids and other components) a major role in synergizing the antiplasmodial activity of artemisinin.

Some important questions, however, remain: (1) how fast are the flavonoids present in these extracts eliminated from the body? (2) would a pharmaceutical preparation containing artemisinin and the bioactive flavonoids, taken 4 times a day for 5–7 days, be more efficient than the tea to clear parasitemia?, (3) how long the effect of flavonoids on CYP450 enzymes would last, and (4) could the recurrence issue be avoided by using the appropriate dosage of artemisinin and flavonoids taken for longer than the 5- to 7-day course recommended? After all we have learned so far about artemisinin and flavonoid extraction and analysis, can we improve the way of making the artemisinin tea to improve its efficacy? For instance if polymethoxylated flavonoids are more bioavailable and bioactive than hydroxylated flavonoids, can hot water extract them or should one add a small percentage (5% or so) of grain alcohol? If the full flowering stage has been reported as the developmental stage highest in those flavonoids, shouldn’t we also investigate the effect of tea made from flowering material? Considering the different biotypes of *A. annua*, the tea should be standardized so that everyone interested could have access to the same tea material, which would be lyophilized and kept frozen and protected from light in the attempt to prevent degradation of its flavonoids. What is currently know, however, does not indicate that the traditional artemisia tea is recommended as a substitute for the ACT, but that it could be valuable in delaying coma until the patient reaches a clinic or hospital stocked with ACT. 

## 14. Conclusions

Although we could not find any studies where the combination of a group of flavonoids (other than from the artemisia tea), specific flavonoids, or at least a crude plant extract was tested in combination with an antimalarial or anticancer drug *in vivo*, recent evidence is that common dietary flavonoids (also found in *A. annua*) such as quercetin, apigenin, luteolin, and kaempferol had both individual and synergistic effects against *Plasmodium falciparum in vitro* [53]. Also, there is evidence that artemisinin was absorbed faster by healthy males who took the *A. annua* tea than by the ones who took pure artemisinin from tablets [7]. Consumption of quercetin, from onions and apples, was also reported as having an inverse association with lung cancer risk [42], which is hypothesized to be due to the flavonoid modulation of CYP450 enzymes, p-glycoproteins, and phase-II enzymes involved in the metabolism of anticancer and other drugs. However, the role of flavonoids also extends to the inhibition of activation of pro-carcinogens, proliferation of cancer cells, apoptosis, and angiogenesis, activation of immune response triggered by cancer cells, and modulation of inflammatory responses [62]. 

Based on the FRAP assay, quercetin was reported to outperform catechins and vitamin E as an antioxidant [178]. These results certainly justify new efforts in the evaluation of flavonoids from *A. annua*, or other plant sources, in combination with artemisinin and its related drugs in the fight against malaria, cancer, or as a plain source of antioxidants. Artemisinin is the most important antimalarial after quinine, but its combination with other drugs is recommended to prevent recurrence and the build-up of drug resistance by *Plasmodium* strains. Although the use of ACT is current in practice, artemisinin has not been tested in combination with flavonoids *in vivo* in humans and, mostly important, in children who have little immunity against malaria and are its major victims. Natural products constitute over 50% of the anticancer arsenal as natural compounds (14%), derivatives (26%), or as synthetic products modeled after the natural model (14%) [179]. However, most are very expensive due to their very low content in the plant, such as vincristine, the plant source having a slow growth such as *Taxus* spp., or the source of the compound being the bark or root of a tree, leading to the destruction of the plant source. *Artemisia annua* can produce from 0.5 to over 1% artemisinin in leaf biomass that can reach at least two tons/ha and can be cultivated in a variety of soils and in a wide range of latitude and altitude, in six months or less [33]. Both artemisinin and flavonoids can be extracted from the same biomass by a combination of ethanol and water at the right temperature more efficiently than with hot water alone (as the traditional tea). Unfortunately, the current method of commercial extraction with hexane focuses only on artemisinin, neglecting all the flavonoids that remain in the extracted by-product. This by-product can be further explored as a rich source of flavonoids or immediately used in animal feed. Lastly, over 2,000 years of traditional use of tea and animal studies with artemisinin have shown that both *A. annua* flavonoids and artemisinin have a wider therapeutic margin than several anticancer natural products currently in use, and without the undesirable side-effects caused by them. Of course, only experimentation will tell if the same benefits and low toxicity of artemisinin observed for the short-term treatment of malaria will also be obtained for its long-term use against cancer. All of these facts taken into consideration certainly indicate that the research of the sesquiterpene lactone artemisinin, combined with *A. annua* flavonoids, as anticancer and anti-parasitic drugs have certainly more to offer than research conducted with either of those natural product classes alone. 

Finally, *A. annua* is an important medicinal plant as a source of artemisinin, currently the most effective natural drug against malaria parasites. Artemisinin also shows anti-proliferative activities against other parasitic organisms (e.g, *Schistosoma* spp.) and cancer cells in animals with the same potential to benefit humans. In ancient China, *A. annua* was used as a tea to treat variety of medical conditions and was considered a preparation that favoured longevity. The artemisinin content in current cultivars is commonly around 1%, and one may wonder if all the medical effects of the traditional *A. annua* tea are solely attributed to artemisinin. The plant is a rich source of polyphenols, and we suspect that these polyphenols contribute to some of the medical benefits of drinking *A. annua* tea. Some polyphenols are known to have their own biological activities, and to synergize common anti-cancer drugs. The research on *A. annua* polyphenols is expected to require a substantial amount of resources due to the number of polyphenols present in the plant and the difficulties associated with their isolation. However, it is worthwhile to look into the great potential interaction between artemisinin and polyphenols. This research, however, will be faster and easier accomplished by an international and multidisciplinary collaboration among physicians, chemists, phytochemists, horticulturists, traditional geneticist, molecular biologists, *etc.*, if it is pursued by different groups, including academia, governmental and non-governmental organizations, interested in making a real contribution in the field of cancer, malaria, and other chronic and parasitic diseases against which artemisinin has shown a promising effect.
